# Broad diversity of bacteria degrading 17ß-estradiol-3-sulfate isolated from river sediment and biofilm at a wastewater treatment plant discharge

**DOI:** 10.1007/s00203-021-02409-0

**Published:** 2021-06-03

**Authors:** Tamara Mainetti, Marilena Palmisano, Fabio Rezzonico, Blaž Stres, Susanne Kern, Theo H. M. Smits

**Affiliations:** 1 Environmental Genomics and Systems Biology Research Group, Institute of Natural Resource Sciences, Zurich University of Applied Sciences (ZHAW), Wädenswil, Switzerland; 2 Faculty of Civil and Geodetic Engineering, University of Ljubljana, Ljubljana, Slovenia; 3 Jozef Stefan Institute, Ljubljana, Slovenia; 4 Environmental Analytics Group, Institute of Chemistry and Biotechnology, Zurich University of Applied Sciences (ZHAW), Wädenswil, Switzerland

**Keywords:** Conjugated estrogens, Biodiversity, Enrichment cultures, Identification

## Abstract

Conjugated estrogens, such as 17β-estradiol-3-sulfate (E2-3S), can be released into aquatic environments through wastewater treatment plants (WWTP). There, they are microbiologically degraded into free estrogens, which can have harmful effects on aquatic wildlife. Here, the degradation of E2-3S in environmental samples taken upstream, downstream and at the effluent of a WWTP was assessed. Sediment and biofilm samples were enriched for E2-3S-degrading microorganisms, yielding a broad diversity of bacterial isolates, including known and novel degraders of estrogens. Since E2-3S-degrading bacteria were also isolated in the sample upstream of the WWTP, the WWTP does not influence the ability of the microbial community to degrade E2-3S.

## Introduction

Estrogenic compounds such as 17β-estradiol (E2) and estrone (E1) can cause alterations in the endocrine system of vertebrates and fish (Hanselman et al. 2003; Reddy et al. 2005; Adeel et al. 2017) in the aquatic environment. The effects of estrogenic compounds on fish include abnormal development of gonads, secondary sexual characteristics and vitellogenin induction (Vermeirssen et al. 2005; Duong et al. 2010; Wang et al. 2012). Estrogenic compounds are suspected to alter the reproduction systems of mussels, birds, reptiles and mammals, which could lead to population decline (do Nascimento et al. 2018). Estrogens can also have negative effects on humans when entering the food chain (Adeel et al. 2017), such as an increased risk of cancer and induce cardiovascular diseases (Wocławek-Potocka et al. 2013).

Wastewater treatment plants (WWTP) were shown as an entry point into the environment of not only the estrogenic compounds like E2 and E1 (Khanal et al. 2006; Ying et al. 2009; Naldi et al. 2016), but also of conjugated estrogens, excreted by humans mainly in the urine, that can all enter the aquatic environment at low ng/L concentrations (D’Ascenzo et al. 2003; Reddy et al. 2005; Kumar et al. 2012; Liu et al. 2015; Ma et al. 2016; Naldi et al. 2016; Ben et al. 2017; Ma and Yates 2018).Glucuronide- or sulfate-conjugated estrogens are more polar than the corresponding free estrogens (Anstead et al. 1997; Fang et al. 2001) and consequently exhibit lower binding affinity for estrogen receptors rendering them biologically inactive (Desbrow et al. 1998; Griffith et al. 2014; Ma and Yates 2018). However, conjugated estrogens may be converted to the free form via hydrolysis by bacterial enzymes (Tyler and Routledge 1998; Bai et al. 2013).

Different studies established that the cleavage of conjugated estrogens depends on the conjugate type. D’Ascenzo et al. (2003) showed that glucuronide conjugates were deconjugated within a WWTP with a higher efficiency of 84–97% compared to 64% for the sulfate conjugate estrone-3-sulfate (E1-3S) (Desbrow et al. 1998). In the study of Isobe et al. (2003), the effluents and the receiving waters of two sewage treatment plants (STP) contained besides free estrogens also E1-3S and E2-3S (17β-estradiol-3-sulfate) but no glucuronide conjugates (Isobe et al. 2003). Comparison of the half-life of glucuronide conjugates and sulfate conjugates in soil showed that sulfate conjugates were more resistant to hydrolysis than glucuronide conjugates (Casey et al. 2020). These results showed that estrogen sulfate conjugates can enter the ecosystem through the effluent of STPs or WWTPs. Subsequent deconjugation in the environment results in free estrogens and other metabolites that can potentially lead to the reported adverse effects on the aquatic ecosystem (Hanselman et al. 2003; Ma and Yates 2018).

The goal of this study was to investigate the degradation potential of E2-3S in rivers and at the outflow of a WWTP. Therefore, microbial communities involved in the degradation of E2-3S in the sediment of the river Murg upstream and downstream of the WWTP Frauenfeld (Switzerland) as well as in the biofilm of the effluent were compared using enrichment cultures combined with terminal restriction fragment length polymorphism (T-RFLP) in this study. In addition, isolation of microorganisms degrading E2-3S was conducted, to identify the responsible microorganisms in enrichment cultures. The results show that E2-3S degradation is present in all tested samples and that a broad diversity of predominantly well culturable organisms was involved in the degradation process.

## Materials and methods

### Enrichment cultures

Samples were taken from the river Murg, north of the city of Frauenfeld (Switzerland) in close proximity of the outflow of the local WWTP. The river Murg has a catchment area of 213 km^2^, a modeled mean annual 4.11 runoff m^3^/s and a pluvial inferior river regime (Federal Office for the Environment 2019, 2020). Samples from the outflow were taken by scraping off the biofilm from the surface of the outflow channel (47.57322 N, 8.89212 E). Sediment samples were taken 450 m upstream (47.56873 N, 8.89443 E) and 520 m downstream the outflow (47.57702 N, 8.88844 E). At each location, three sediment samples were taken across the width of the river and combined after sieving through a 2 mm sieve to get a representative sample of the sediment. Detailed analysis of microbial biogeography was not within the scope of this study.

**Fig. 1:**
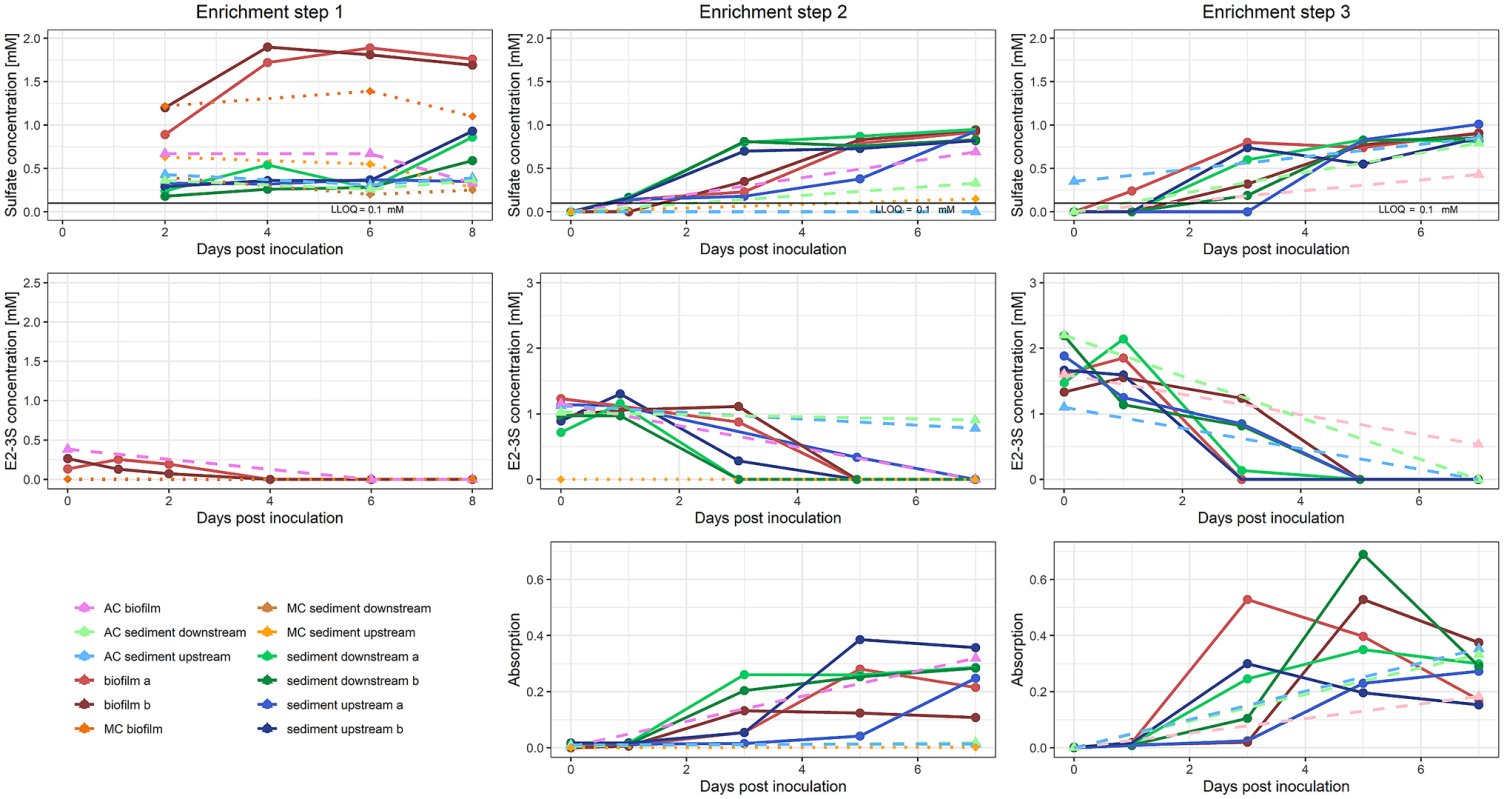
Substrate and product profiles of the three enrichment steps (shown from left to right). Top row: sulfate concentration analyzed by the turbidimetric method; middle row: E2-3S concentration analyzed by LC-Q-TOF; and bottom row: absorption measurements at 600 nm (OD600). The LLOQ (0.1 mM) of the sulfate determination is marked with a black horizontal line. Solid lines are the samples, dashed lines are the matrix control (MC, in orange) and the autoclaved controls (AC1-3) of the corresponding samples

Duplicate enrichment cultures (indicated in Fig. 1 with an “a” and “b”, respectively) were prepared in 250 ml baffled Erlenmeyer flasks containing 50 ml of a low-sulfate phosphate-buffered mineral-salts medium, pH 7.2 (Thurnheer et al. 1986). After autoclaving, the substrate 17β-estradiol-3-sulfate (E2-3S) sodium salt (Toronto Research Chemicals, Toronto, Canada) was added in solid form at a final concentration of 1 mM (18.72 mg per culture) and shaken until no more crystals were visible. The first enrichment step was started by adding 10 g of sediment or biofilm, later steps by transferring 0.5 ml of the culture volume to 50 ml of fresh medium. Incubation was done on an orbital shaker at 28 °C. Autoclaved control cultures (soil or biofilm samples autoclaved once 20 min at 120 °C) for each sampling point were also included in the experiment and treated as the other samples. Matrix control cultures (minimal medium with soil or biofilm of the respective samples without E2-3S) were added in the first enrichment step to check for growth in absence of the substrate E2-3S. In these cultures, only sulfate was measured. The matrix control in enrichment step 2 consisted of 50 ml carbon-limited minimal medium without E2-3S.

From the cultures, aliquots of 4 ml were taken after 24 h followed by 48 h intervals to measure microbial growth and to determine the concentrations of the substrate E2-3S and the product sulfate. As a blank for the optical density at 600 nm (OD_600_), the mineral medium without E2-3S was used. In enrichment step 1, the measurement of the OD_600_ was not possible due to the high amount of fine sediment and biofilm material in the sample, causing a high turbidity. The sulfate concentration during the enrichment was determined using a turbidimetric method as described before (Kolmert et al. 2000). After the second and third passage in fresh medium, a single inoculation loop (approx. 5 ml) from each culture was streaked on Luria–Bertani (LB) agar, and single colonies showing different morphologies were picked to fresh LB medium. Single colonies were then tested for growth in tubes containing 5 ml of minimal medium with 1 mM E2-3S. Colonies obtained after plating out with an inoculation loop from these cultures were used for microbial identification through 16S rRNA gene sequencing.

The aliquots taken from the enrichment cultures were centrifuged for 1 min at 10,000 rpm to remove cells and particulate matter. The supernatant was diluted appropriately in 10 mM acetate/acetonitrile (95:5, v/v) to be within the detection range and then filtered with a 0.45 µm filter, to remove particles. The quantitative analysis of E2-3S degradation was performed with the UHPLC System 1290 Series coupled to a 6540 UHD Accurate-Mass Q-TOF LC/MS from Agilent Technologies (Santa Clara, CA, USA) with an electrospray ionization (ESI) in the negative ion mode. The separation was performed at 30 °C on an Accucore™ C18 column (2.1 × 100 mm, 2.6 µm) from Thermo Fisher Scientific (Waltham, MA, USA). The mobile phase consisted of (A) 10 mM ammonium acetate (Sigma-Aldrich, Buchs, Switzerland) in water (pH ~ 6.5) and (B) acetonitrile (Sigma-Aldrich). The gradient used was as follows: after applying 5% solvent B at 0 min to 2 min, a linear gradient to 95% solvent B at 6 min was implemented, followed by 1 min hold at 95% solvent B. Afterward, a linear gradient back to 5% solvent B at 8 min was followed by an hold for 1 min. The injection volume and the flow rate were 10 µl and 0.5 ml/min, respectively. Detection was made with a 1290 Infinity Diode Array Detector (DAD) at 281 nm and high-resolution mass spectrometry in full scan. The level of detection (LOD) for the Q-TOF was 1 μg/l while the lower level of quantification (LLOQ) was set to 3 μg/l. The upper level of quantification (ULOQ) was 100 μg/l.

### Terminal restriction fragment length polymorphism (T-RFLP) analysis

DNA extraction of the original samples and the samples taken at the end of enrichment steps 1, 2, and 3 was performed using the DNeasy PowerLyzer PowerSoil Kit (QIAGEN, Hilden, Germany) according to the manufacturer’s instructions. The partial 16S rRNA gene was amplified from DNA extractions by PCR using fluorescently labeled primers for bacteria [forward 8F_Red (5ʹ-AGA GTT TGA TCC TGG CTC AG-3ʹ) with fluorophore AT565 and reverse 534R_Green (5ʹ-ATT ACC GCG GCT GCT GGC-3ʹ) (Lane 1991; Muyzer et al. 1993) with fluorophore AT532] (Microsynth AG, Balgach, Switzerland). The DNA Polymerase KAPA2G Robust HotStart ReadyMix (KapaBiosystems Wilmington, Massachusetts, United States) was used according to manufacturer’s instructions. PCR amplifications were carried out as described before (Schmautz et al. 2021) on a T100 Thermocycler (Bio-Rad Laboratories, Inc., Hercules, California, United States). Products of the PCR were end-treated for the correction of the overhanging ends using Klenow polymerase (Osborn et al. 2000) and were cleaned with a Millipore MultiScreen PCRμ96 filter plate (Merck KGaA, Darmstadt, Germany). Finally, the products were resuspended in 25 μl PCR-grade water. Purified PCR amplicons were digested using the restriction enzyme *Alu*I (New England Biolabs, Bioconcept, Allschwil, Switzerland) according to the manufacturer’s instructions. Each 1 μl of digestion product was mixed with 18.65 μl Hi-Di formamide and 0.35 μl GeneScan LIZ 600 Size Standard (Thermo Fisher Scientific, Massachusetts, United States), denatured and analyzed using an ABI 3500 capillary sequencer (Thermo Fisher Scientific).

### T-RFLP data analysis

Profiles obtained with T-RFLP were analyzed using the GeneMapper^®^ Software 5 (Applied Biosystems, Thermo Fisher Scientific, Massachusetts, United States). Restriction fragments between 40 and 500 base pairs were included in the analysis and exported as raw data. Further data processing was carried out using the software T-REX (Culman et al. 2009). For this, terminal restriction fragments (T-RFs) between 40 and 500 bp and a peak height exceeding 150 RFU were selected. To characterize microbial diversity, Shannon’s diversity and Simpson’s dominance indices were calculated (Hill et al. 2003). For the statistical evaluation of the abundance matrix generated from the T-REX software, a non-metric multidimensional scaling (NMDS) was performed using the function metaMDS of the R-package vegan (Oksanen et al. 2019). The distance was calculated using the Bray–Curtis index (Bray and Curtis 1957). The representation of the initial matrix in the low-dimensional space is expressed by the stress value (Holland 2008).

### Identification of E2-3S degrading microbial isolates

Chromosomal DNA was obtained by taking a dilute sample of an overnight culture, heat for 10 min at 95 °C. The supernatant was used as a template for PCR. For 16S rRNA sequencing, a fragment (around 900 bp) of the 16S rRNA gene of the different isolates was amplified by PCR using the primers 16S_p10-27_f (5ʹ-AGT TTG ATC MTG GCT CAG-3ʹ) and 16S_p942-927_r (5ʹ-ACC GCT TGT GCG GGC C-3ʹ) (Lane 1991). Amplicons were sent to Microsynth AG for Sanger sequencing. The obtained sequences were assembled with the program SeqMan Pro (version 12.1.01; DNASTAR, Madison, WI, USA) using standard settings. The assembled data were identified using the BLAST search tool (Altschul et al. 1990) using the non-redundant database and standard settings. For those 16S rRNA gene sequences of bacteria that could only be identified on genus level, the sequences were compared with reference sequences of the type strains of the same genus, downloaded for the respective genus from the LPSN webpage (https://www.bacterio.net/), to identify the bacteria on species level. To align the data and to generate phylogenetic trees, the software MEGA X ((Kumar et al. 2018), version 10.1.6) was used. The evolutionary history was inferred using the Maximum Likelihood method (Tamura et al. 2012). Initial tree(s) for the heuristic search were obtained automatically by applying Neighbor-Join and BioNJ algorithms to a matrix of pairwise distances estimated using the Maximum Composite Likelihood (MCL) approach, and then selecting the topology with superior log likelihood value. Bootstrap test (1000 replicates) was used to calculate the percentage of replicate trees in which the associated taxa were clustered together (Felsenstein 1985). 

## Results

### Enrichment cultures

Three subsequent rounds of enrichment in duplicate cultures with sediment or biofilm sample in a low-sulfate minimal medium (Thurnheer et al. 1986) with E2-3S as a sole carbon and energy source were performed. In the first enrichment step, we followed sulfate concentration as indication for substrate utilization in all cultures and E2-3S concentration in biofilm samples, whereas in later enrichment steps, we also included E2-3S concentration for the other cultures (Fig. 1), as sediment particles were reduced after the first transfer. Sulfate concentrations in the first enrichment step increased only slightly in most cultures but increased for about 1 mM in three cultures (Fig. 1), which would correspond to the amount of E2-3S added to the culture. The higher background could be caused by the presence of sulfate in the inoculum, or from degradation of other sulfur-containing substrates. In the two later enrichment steps, a higher concentration of sulfate was not observed anymore. E2-3S concentrations in enrichment steps 2 and 3 dropped down to below detectable levels within the time of incubation, while stoichiometric concentrations of sulfate were obtained (Fig. 1). This shows that the enriched organisms are at least able to completely deconjugate E2-3S, and, as it is the sole carbon source in the cultures, also must be able to (partly) use the compound as a carbon source.

The sulfate concentrations in two out of three autoclaved control cultures also showed an increase in the second enrichment step, while substrate concentrations were decreasing over time. However, only in the autoclaved biofilm control culture, the OD_600_ was increasing, whereas in the sediment samples, no increase in OD_600_ was observed, indicating only limited amounts of growth. It cannot be excluded that part of the decrease in substrate concentration is due to chemical decay of the substrate. In the third enrichment step, all three cultures showed growth to similar levels as in the sediment or biofilm cultures, increase in sulfate concentration and decrease of E2-3S concentration. This would indicate that some bacteria must have survived the autoclaving process, which are able to grow with E2-3S as a carbon source.

### Microbial diversity in the cultures

T-RFLP analysis were conducted to explore different alpha-diversity parameters of the original samples accessible through fast fingerprinting of the microbial communities. These analyses showed that both original sediment samples had a lower Shannon index and taxa richness than the original biofilm sample, whereas the Simpson’s index (evenness) of all original samples was similar. Analysis of the alpha-diversity parameters for the individual enrichment steps did not give conclusive data, as there was no detectable decrease of the alpha-diversity due to the enrichment of specific degraders (Table 1).

**Table 1:** Alpha-diversity parameters based on the peak patterns in T-RFLP

Source	Sample	Step	Shannon index	Evenness	Taxa richness
Upstream	Sediment		3.54	0.88	57
	Culture 1	1	3.23	0.83	48
		2	3.21	0.76	70
		3	3.20	0.75	70
	Culture 2	1	3.22	0.74	79
		2	3.41	0.74	98
		3	3.54	0.78	77
Biofilm	Biofilm		3.71	0.87	71
	Culture 1	1	3.59	0.81	84
		2	3.09	0.70	82
		3	3.37	0.77	77
	Culture 2	1	3.38	0.83	58
		2	3.01	0.80	42
		3	3.43	0.77	79
Downstream	Sediment		3.62	0.88	62
	Culture 1	1	3.40	0.75	92
		2	3.17	0.77	60
		3	2.83	0.69	61
	Culture 2	1	3.46	0.83	65
		2	3.41	0.78	77
		3	3.21	0.71	92

**Fig. 2:**
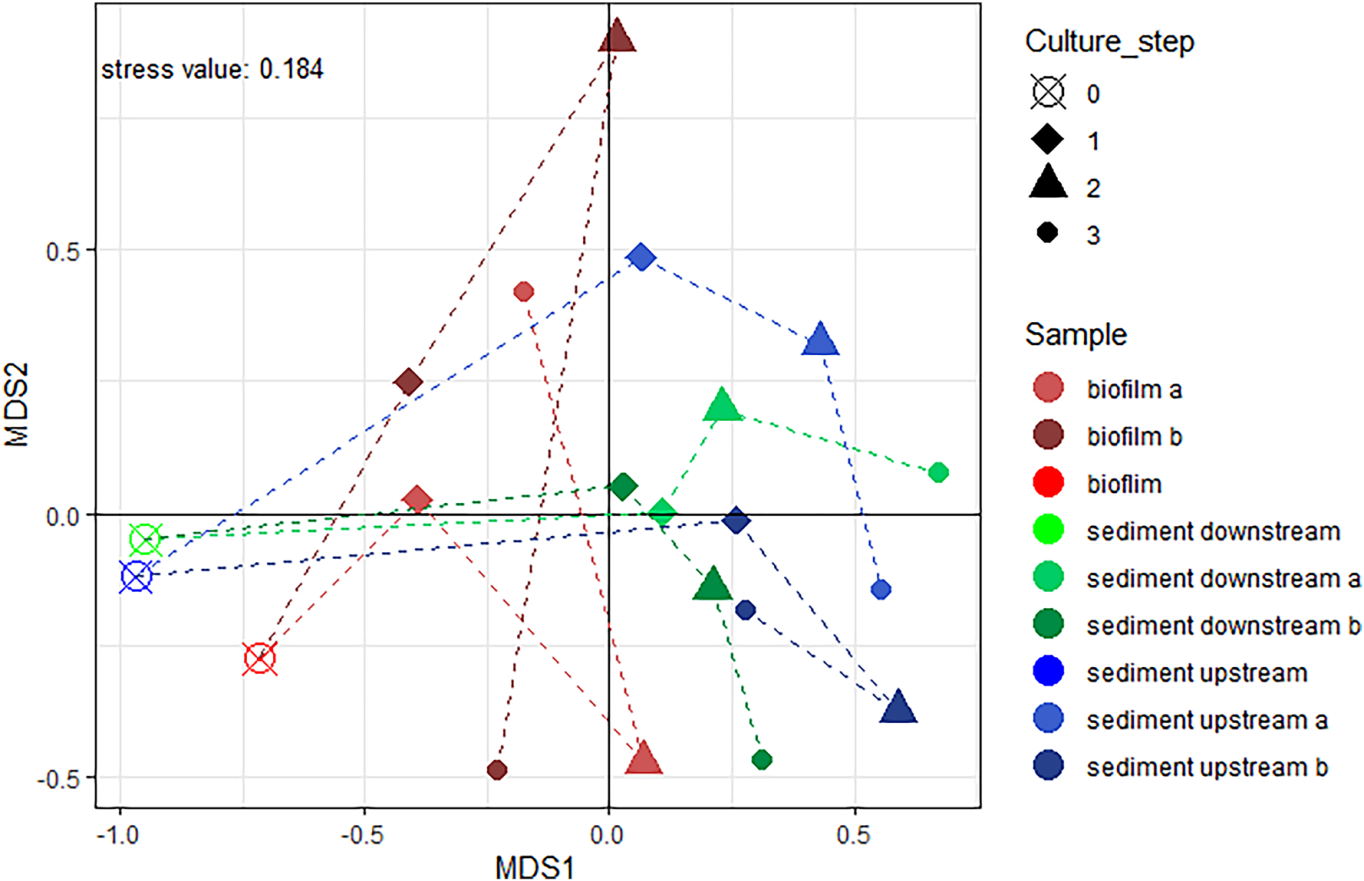
NMDS of all samples over the whole enrichment and the corresponding original samples with a stress value of 0.184

The non-metric multidimensional scaling (NMDS) analysis of the community structures (Fig. 2) provided, with a stress value of 0.184, a sufficient representation of the data in the low-dimensional space. It showed that the community structures of the original sediment and biofilm samples were similar to each other. Throughout the whole enrichment, the samples originating from the sediments were more similar to each other and showed a larger dissimilarity to the original sample than the samples originally from the biofilm.

### Isolation and identification of E2-3S-degrading bacteria

At the end of enrichment enrichment steps 2 and 3, samples were streaked from all different cultures onto LB medium and individual colonies further purified by restreaking. The isolates were first tested for growth in liquid minimal medium with E2-3S to test for their ability to grow with this compound. The isolates that had an increased turbidity in presence of E2-3S were stored and used for further work.

**Fig. 3:**
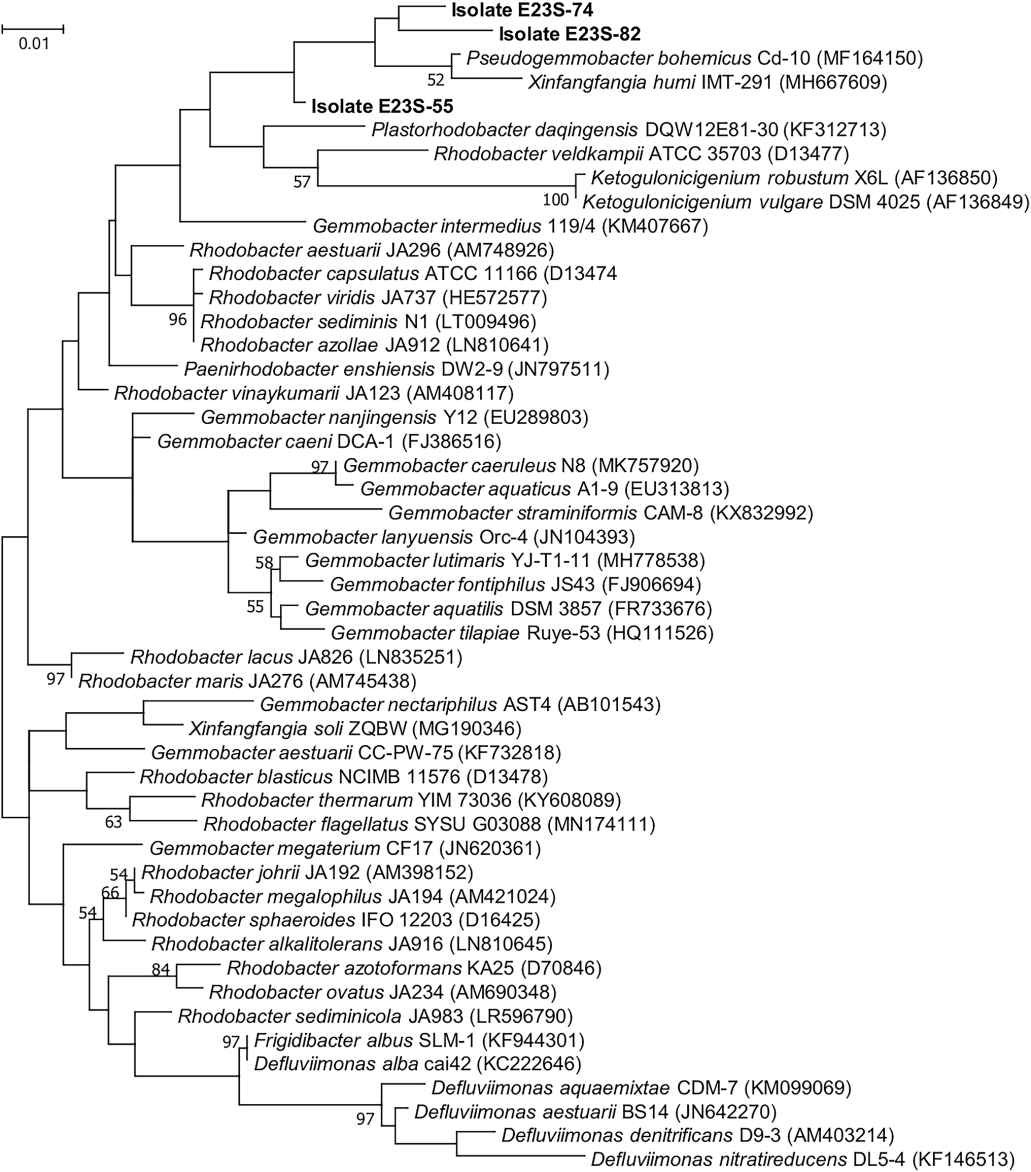
Maximum Likelihood tree of the three *Rhodobacteriaceae* isolates together with all type strains of the *Rhodobacteriaceae* (Genbank accession number in brackets). All positions containing gaps and missing data were eliminated. There was a total of 710 positions in the final dataset. Bootstrap values over 50% are indicated next to the branches

In total, 26 isolates could be identified using partial 16S rRNA gene sequencing (Table 2). Of these isolates, 11 were isolated from cultures that originated upstream of the WWTP, seven from the outflow biofilm and 8 from downstream the WWTP. Several of the isolates from enrichment step 2 were also identified from the isolates of enrichment step 3. The tentative identifications to type strains of the respective genera showed that three isolates (E23S_55, E23S_74 and E23S_82), all belonging to the *Rhodobacteriaceae*, could not be identified at the genus level. From a Maximum Likelihood tree with an alignment of the 16S rRNA genes of all type strains of the surrounding genera (Fig. 3), it was not possible to assign these isolates to either the genus of *Pseudogemmobacter* or to *Xinfangfangia*. All other isolates could at least be appointed to the genus level, and several even to the species level as the pairwise distances to the next species were large enough. However, the identification should be improved when further work is done with the isolates.

**Table 2:** Isolates obtained in this study and result of tentative identification using BLASTN hits to reference sequences of bacterial type strains

Step	Isolate	Source^a^	Closest type strain hit			Tentative identification	
			% Identity	To type strain	GenBank Acc. no	Family	Species
3	E23S_54	Upstream	99.5	*Pseudomonas mendocina* ATCC 25411^T^	NR_114477.1	*Pseudomonadaceae*	*Pseudomonas mendocina*
	E23S_55	Upstream	96.7	*Pseudogemmobacter bohemicus* Cd-10^T^	MF164150.2	*Rhodobacteraceae*	n.d.^b^
	E23S_56	Upstream	99.9	*Empedobacter brevis* LMG 4011^T^	AM177497.1	*Flavobacteriaceae*	*Empedobacter brevis*
	E23S_57	Outflow	99.9	*Acinetobacter oryzae* B23^T^	MH071139.1	*Moraxellaceae*	*Acinetobacter oryzae*
	E23S_58	Outflow	99.1	*Microbacterium esteraromaticum* DSM 8609^T^	Y17231.1	*Microbacteriaceae*	*Microbacterium* sp.
	E23S_59	Downstream	99.8	*Empedobacter brevis* LMG 4011^T^	AM177497.1	*Flavobacteriaceae*	*Empedobacter brevis*
	E23S_60	Downstream	100	*Acinetobacter towneri* DSM14962^T^	EF611416.1	*Moraxellaceae*	*Acinetobacter towneri*
	E23S_61	Downstream	99.6	*Delftia acidovorans* LMG 1226^T^	EU024145.1	*Comamonadaceae*	*Delftia acidovorans*
	E23S_62	Upstream	99.2	*Pseudomonas japonica* NBRC 103040^T^	AB681920.1	*Pseudomonadaceae*	*Pseudomonas japonica*
	E23S_63	Outflow	99.2	*Pseudomonas japonica* NBRC 103040^T^	AB681920.1	*Pseudomonadaceae*	*Pseudomonas japonica*
	E23S_65	Downstream	99.2	*Pseudomonas japonica* NBRC 103040^T^	AB681920.1	*Pseudomonadaceae*	*Pseudomonas japonica*
2	E23S_66	Upstream	99.6	*Alcaligenes faecalis* subsp. *faecalis* NBRC 13111^T^	AB680368.1	*Alcaligenaceae*	*Alcaligenes faecalis*
	E23S_67	Upstream	99.9	*Bacillus subtilis* ATCC 6051^T^	JF749278.1	*Bacillaceae*	*Bacillus* sp*.*
	E23S_68	Upstream	97.5	*Sphingopyxis ummariensis* UI2^T^	EF424391.2	*Sphingomonadaceae*	*Sphingopyxis* sp.
	E23S_69	Upstream	99.5	*Bordetella trematum* LMG 13506^T^	KF601906.1	*Alcaligenaceae*	*Bordetella trematum*
	E23S_70	Upstream	100	*Rhodococcus defluvii* Ca11^T^	KC788572.1	*Nocardiaceae*	*Rhodococcus defluvii*
	E23S_71	Upstream	96.8	*Sphingobacterium alimentarium* DSM 22362^T^	FN908502.1	*Sphingobacteriaceae*	*Sphingobacterium* sp.
	E23S_72	Upstream	96.9	*Comamonas aquatica* LMG 2370^T^	AJ430344.1	*Comamonadaceae*	*Comamonas* sp.
	E23S_73	Outflow	99.1	*Pseudomonas japonica* NBRC 103040^T^	AB681920.1	*Pseudomonadaceae*	*Pseudomonas japonica*
	E23S_74	Outflow	97.8	*Pseudogemmobacter bohemicus* Cd-10^T^	MF164150.2	*Rhodobacteraceae*	n.d
	E23S_75	Outflow	99.5	*Pseudomonas hunanensis* LV^T^	JX545210.1	*Pseudomonadaceae*	*Pseudomonas* sp.
	E23S_76	Outflow	99.5	*Kaistia adipata* DSM 17808^T^	AY039817.1	*Rhizobiaceae*	*Kaistia adipata*
	E23S_79	Downstream	99.4	*Comamonas testosteroni* LMG 1800^T^ = ATCC 11996^T^	EU024141.1	*Comamonadaceae*	*Comamonas testosteroni*
	E23S_82	Downstream	97.2	*Pseudogemmobacter bohemicus* Cd-10^T^	MF164150.2	*Rhodobacteraceae*	n.d
	E23S_85	Downstream	99.6	*Alcaligenes faecalis* subsp. *faecalis* NBRC 13111^T^	AB680368.1	*Alcaligenaceae*	*Alcaligenes faecalis*
	E23S_87	Downstream	99.3	*Alcaligenes faecalis* subsp. *faecalis* NBRC 13111^T^	AB680368.1	*Alcaligenaceae*	*Alcaligenes faecalis*

^a^Upstream: sediment upstream of the WWTP; outflow: biofilm at outflow of the WWTP; downstream: sediment downstream of the WWTP

^b^n.d.: not determinable. See text for further explanations

## Discussion

### Presence of bacteria able to grow with E2-3S

The enrichment procedure used in this study yielded a set of isolates that showed the ability to grow on E2-3S as sole carbon source. Therefore, it is proposed that the natural community at the sampled environments contains a diverse population of bacteria that are able to grow with E2-3S. The bacterial community in the effluent of the WWTP Frauenfeld may thus not influence the population of bacteria growing with E2-3S significantly in the river Murg.

It was reported that animal waste, in particular animal urine (Hoffmann et al. 1997; Isobe and Shimada 2003), can be a source of estrogen sulfates in the environment, especially from grazing livestock or land-application of animal waste (Scherr et al. 2009). Upstream of the WWTP Frauenfeld are various fields, pasture and grassland for agriculture use, which could be a source of E2-3S. This would explain the presence of populations in all samples, independently from the sampling side that are able to grow with E2-3S as a carbon source. It would require a more systematic sampling of the specific populations, the quantities of each potential degrader and environmental concentrations of sulfonated estrogens to examine the role of these organisms in natural degradation thereof.

### Identification of isolates

Many isolates obtained in this study were identified to the species level based on partial 16S rRNA gene sequence comparisons. Some of them, like *Rhodococcus* spp., *Pseudomonas* spp. and *Alcaligenes* spp. (Payne and Talalay 1985; Slezack-Deschaumes et al. 2012; Cregut et al. 2013), include known E2-3S degraders (Scherr et al. 2009; Zheng et al. 2013; Ma and Yates 2017, 2018). Some others have close relatives that were detected in other studies, while also novel organisms were identified as E2-3S degraders. Genera like *Acinetobacter*, *Empedobacter* or *Kaistia* were as such not yet associated to steroid degradation in previous studies.

Six isolates were assigned to the genus *Pseudomonas*, belonging to three different taxonomic units. Generally, it is not possible to only base the identification of *Pseudomonas* spp. from the 16S rRNA gene level, as the levels of sequence identities of this gene are very close (Mulet et al. 2012). Therefore, the use of other identification methods such as multiple locus sequence analysis (MLSA) and/or genome sequencing should be applied to give a definite assignment to the respective species (Frasson et al. 2017; Rutz et al. 2019) and insight into their genetic makeup.

### Potential degradation pathways

The pathway for degradation of E2-3S most probably proceeds via the initial reduction of the substrate to E1-3S. We were unable to detect any peaks in the growth medium at the position of E2 reference material using measurements with the Diode Array Detector (DAD) at wavelength 281 nm. This excludes the initial deconjugation of E2-3S to E2 as a potential mechanism and would support the data of previous studies (Scherr et al. 2009; Ma and Yates 2017, 2018), in which E1, the deconjugated product of E1-3S, is reported as intermediate. However, this pathway cannot be confirmed within this study, since E1 was not analyzed.

Although several pathways are possible for the degradation of steroid compounds, it is expected that at least several of the isolates degrade E1 through the recently identified aerobic 4,5-seco pathway (Wu et al. 2019). We did not investigate whether the dead-end product of this pathway (pyridinestrone acid) was accumulating in the cultures (Chiang et al. 2020). The final product of the 4,5-seco pathway is 3aα-H-4α(3ʹ-propanoate)-7aβ-methylhexahydro-1,5-indanedione (HIP). Chiang et al. (2020) showed that the genomes of most of the verified steroid degrading bacteria harbor gene clusters potentially involved in the HIP degradation pathway (Chiang et al. 2020). These gene clusters were found, among others, in different *Pseudomonas* spp., *Comamonas testosteroni* and *Rhodococcus* spp. (Chiang et al. 2020). These findings led to the assumption that the isolates characterized as *Pseudomonas* spp., *C. testosteroni* and *Rhodococcus* spp. in this study are likely to contain the gene clusters involved in the HIP degradation as well.

### Autoclaved controls

All conducted analyses were indicating that the autoclaved sediment and biofilm material was not sufficiently sterilized. After the confirmation of growth on E2-3S, each autoclaved control contained at least one colony type, for which the isolate was identified to belong to the genus *Pseudomonas*. Different reasons for this contamination of the autoclaved controls are possible. One reason could be a contamination during the first inoculation, which is relatively unlikely as growth in the first cultures was largely absent. Another option is that the autoclaving of the sediment and biofilm sample was not sufficient to sterilize the controls and therefore showed some biological activity. Similar effects were already reported before, even with autoclaving sediment three times (Tuominen et al. 1994; Otte et al. 2018). The survival of certain bacteria can be explained by various survival strategies, such as reduced metabolism in dormant state, and different repair and resistance mechanisms (Otte et al. 2018). Another aspect of autoclaving to consider is that autoclaving sediment leads to a release of nutrients and substrate (Tuominen et al. 1994; Shaw et al. 1999; Otte et al. 2018). This increase could be beneficial for the bacteria that survived autoclaving. This may explain the degradation of E2-3S only after the second round of cultivation.

### Conclusions

The enrichment strategy to obtain bacteria able to grow with E2-3S as sole carbon source adopted in this study was successful, even though the enrichment cultures still had a large diversity as seen from the diversity indices. It nevertheless showed that there are, independent of the sampling site, E2-3S-degrading bacteria present in the watershed of the river Murg. It is not clear whether the E2-3S-degrading bacteria occur in the environment as a result of artificial E2-3S sources, like WWTP or animal farming, and hence necessitate further investigations to identify potential E2-3S sources but also the actual degradation pathways.

The broad diversity of the isolates obtained in this study using relatively simple and accessible general microbiology approaches allows us now to assess the diversity of biodegradation pathways in these isolates through genome sequencing. Based on available information from relatives with published genomes, it can be envisaged that the genes encoding the 4,5-seco pathway will be found in the genomes of several isolates (Bergstrand et al. 2016; Chiang et al. 2020), while variant or even novel pathways may be identified for other isolates obtained, for which the species have never been associated with estrogen degradation. This will enable us to create diagnostic primers to measure the transcriptional activity of key genes of estrogen degradation in suitable environmental samples as a measure of the pollution of natural water samples by estrogenic compounds.

## Data Availability

The strains isolated, and datasets generated and analyzed during the current study, are available from the corresponding author on reasonable request.

## Code availability

Software used in this study is freely available.
